# Adjuvant chemotherapy for soft tissue sarcomas: a 10-year mono-institutional experience

**DOI:** 10.1007/s00432-015-2065-4

**Published:** 2015-11-07

**Authors:** Antonella Brunello, Mario Domenico Rizzato, Marco Rastrelli, Anna Roma, Marco Maruzzo, Umberto Basso, Pasquale Fiduccia, Maria Samaritana Buzzaccarini, Giovanni Scarzello, Carlo Riccardo Rossi, Vittorina Zagonel

**Affiliations:** 1 Medical Oncology 1 Unit, Istituto Oncologico Veneto IOV – IRCCS, Via Gattamelata 64, 35128 Padua, Italy; 2 Radiation Therapy Unit, Istituto Oncologico Veneto IOV – IRCCS, Padua, Italy; 3 Melanoma and Sarcoma Unit, Istituto Oncologico Veneto IOV – IRCCS, Padua, Italy; 4 Clinical Trials and Biostatics Unit, Istituto Oncologico Veneto IOV – IRCCS, Padua, Italy

**Keywords:** Adjuvant, Sarcoma, High risk, Chemotherapy, Epirubicin, Ifosfamide

## Abstract

**Purpose:**

The role of adjuvant chemotherapy (ACT) for soft tissue sarcomas (STS) is not standard practice. We investigated effectiveness and tolerability of ACT in patients (pts) with operated high-risk STS in clinical practice.

**Methods:**

Medical records of pts with localized STS referred to Istituto Oncologico Veneto, Padova, from January 1, 2003 to July 07, 2012 were reviewed. Data were collected for pts with high-risk STS (size ≥5 cm, high grade and stage III). For those who received ACT, regimens used, drug doses, number of cycles, toxicity, and reasons for dose reduction or treatment interruption were recorded. Disease-free survival (DFS) and overall survival (OS) were calculated with the Kaplan–Meier method.

**Results:**

Out of 96 eligible pts, median age 62 years, 36 received ACT after loco-regional treatment. Median DFS was 29.6 months (95 % CI 13.2–46.0) in pts receiving ACT and 7.8 months (95 % CI 3.9–11.7) in untreated pts (*p* < 0.0001); median OS was 67.0 months (95 % CI 25.4–108.6) in treated and 33.7 months (95 % CI 23.3–44.2) in untreated pts (*p* = 0.005). Among pts receiving ACT, a significant difference in DFS was observed between pts with limb/girdle disease (median DFS 82.4 months; 95 % CI 0.0–184.7) and pts with other primary sites (median DFS 18.3 months; 95 % CI 8.0–28.5) (*p* = 0.052). Grade ≥3 toxicities occurred in 20 pts (20.8 %), leading to dose reductions, delays, and treatment discontinuation in five cases. There was no treatment-related death.

**Conclusion:**

Our data confirm benefit of ACT with regard to DFS and OS in pts with high-risk STS, greatest for limb/girdle STS.

## Introduction

Soft tissue sarcomas (STS) are a heterogeneous group of malignancies which derive from mesenchymal tissue (Clark et al. 2005). STS are rare tumors, which globally make up about 1 % of all malignancies with an overall incidence of 3–5 cases/100.000 inhabitants/year (Siegel and Naishadham 2013). The group includes more than 70 histological subtypes (Mastrangelo et al. 2012) with different natural history, age at the diagnosis, and site of onset. STS arising in the limbs and girdles are by far the most frequent, accounting up to 75 % of the total (Fletcher et al. 2013).

The prognosis may vary among different histotypes, with the most important prognostic factors being tumor size, grade, and depth (Coindre et al. 1996). Some tools, such as the detection of sarcoma circulating tumor cells, are currently under investigation, and they may provide a way to monitor risk of relapse and metastatic spread, which might play a role in the future in clinical decision-making, if validated in large clinical studies (Satelli et al. 2014).

The treatment of soft tissue sarcomas relies on a multi-modality approach, with surgery representing the cornerstone (ESMO/European Sarcoma Network Working Group 2012), combined with ancillary therapies such as radiotherapy or hyperthermic isolated limb perfusion, which allow to obtain an optimal local control of the disease minimizing the need for demolitive procedures (Rosenberg et al. 1982; Rossi et al. 2003).

Nonetheless, a great proportion of patients with high-risk sarcoma eventually develop metastatic disease (Zagars et al. 2003).

This has prompted the conduction of trials with the aim of testing the role of adjuvant chemotherapy in preventing relapse and eventually improving survival. A first generation of trials in the late 1970s tested the efficacy of anthracyclines, alone or in various combination regimens, with discordant findings. The Sarcoma Meta-Analysis Collaboration (SMAC) (1997), which included patients’ individual data from such trials, showed a significant advantage in terms of disease-free survival (DFS) in patients who received adjuvant chemotherapy but no difference with regard to overall survival (OS). Yet, in the subgroup of patients with STS of the extremities, a significant benefit in OS was observed for patients receiving chemotherapy. A second generation of randomized trials was run in the early 1990s, characterized by more rigorous selection criteria, by the introduction of ifosfamide and the intensification of doses with the support of hematopoietic growth factors. This generation of studies yielded discordant results, with a study of the Italian Sarcoma Group demonstrating a benefit both in DFS (*p* = 0.04) and OS (*p* = 0.03) (Frustaci et al. 2001) for patients receiving adjuvant chemotherapy and, on the other hand, a European Organization for Research and Treatment of Cancer (EORTC; Woll et al. 2012) trial showing no difference in OS (*p* = 0.72) and DFS (*p* = 0.51) between patients treated with adjuvant chemotherapy and controls. A new meta-analysis confirmed a marginal efficacy of adjuvant chemotherapy, with a significant decrease in local recurrence rate (OR 0.73, *p* = 0.02), distant recurrence rate (OR 0.67, *p* = 0.001), and overall recurrence rate (OR 0.67, *p* = 0.0001) in patients receiving chemotherapy with doxorubicin-based regimens or with doxorubicin/ifosfamide combination, which translated into a gain in OS (OR 0.77, *p* = 0.01) and absolute risk reduction of death of 6 %, when only doxorubicin plus ifosfamide-based regimens were considered (Pervaiz et al. 2008). The more recently pooled analysis of the two EORTC phase III trials (Le Cesne et al. 2014) conducted on individual patient data of 819 patients with median follow-up of 8.2 years shows that tumor size, high histological grade, and R1 resection are independent adverse prognostic factors for relapse-free survival (RFS) and OS, whereas adjuvant chemotherapy is an independent favorable prognostic factor for RFS but not for OS. Moreover, gender and age correlate with RFS, with males and patients >40 years having a significantly better RFS in the treatment arms, and on the contrary female gender and age <40 years being associated with a marginally worse OS. Patients with R1 resection had a significantly better RFS and OS favoring adjuvant CT arms.

We planned our study in order to evaluate the efficacy and safety of adjuvant chemotherapy in patients with high-risk sarcomas in patients treated at our Institution in common clinical practice, outside clinical trials.

## Methods

Medical records of patients with non-metastatic STS referring to the Units of Medical Oncology 1 and 2 of the Istituto Oncologico Veneto of Padua from January 1, 2003 to July 31, 2012 were reviewed.

All patients aged ≥18 years with high-risk STS, defined as tumor size >5 cm, deep location, high grade, and stage III of the classification of the American Joint Committee on Cancer (AJCC) 2010 (Edge et al. 2010), were considered eligible. STS with specific histologic types such as pediatric-type sarcomas (i.e., rhabdomyosarcoma, Ewing sarcoma), GISTs, and carcinosarcomas were not considered for this study.

Data collected included histological subtype, performance status (PS; Oken et al. 1982), other local therapies (radiotherapy, hyperthermic isolated limb perfusion), and type of surgery. With regard to patients receiving adjuvant chemotherapy, chemotherapy regimen, drug doses, and number of cycles, along with toxicity and reasons for dose reduction or treatment interruption were recorded. Toxicities were evaluated according the Common Terminology Criteria for Adverse Events version 3.0 (CTCAE v3.0; Trotti et al. 2003).

DFS, defined as the time between surgery and disease local or metastatic recurrence, and OS, defined as the time between surgery and death for any cause, were evaluated. For patients lost at follow-up, survival was obtained by consulting the demographic services of patient’s city of residence, with disease status being censored for DFS. For this type of study, formal consent was not required.

Statistical analysis was performed using SPSS software (SPSS for Windows, version 15.0. Chicago, SPSS Inc.) For the estimates of survival, we used Kaplan–Meier product-limit method, comparisons between groups were performed using the log-rank test, and hazard ratio (HR) was calculated using the Cox regression; the association between categorical and ordinal variables was assessed by means of the *X* ^2^ test and the Mann–Whitney *U* test, respectively.

## Results

Out of 331 patients with STS, 96 patients were eligible. Of these, 36 received adjuvant chemotherapy (ACT). Sixty patients did not receive adjuvant treatment (NACT).

### Patients’ characteristics

The most frequent histological subtypes both in the ACT and in the NACT group were leiomyosarcoma (22.2/21.7 %), liposarcoma (22.2/26.7 %), undifferentiated pleomorphic sarcoma (19.4/16.7 %), synovial sarcoma (11.1/8.3 %), and malignant peripheral nerve sheath tumors (8.3/6.7 %). Patients’ characteristics are reported in Table 1.

**Table 1:** Patients’ characteristics

	ACT (%)	NACT (%)
Age		
<65 years	30 (83.3)	26 (43.3)
≥65 years	6 (16.7)	34 (56.7)
Gender		
Male	16 (44.4)	33 (55)
Female	20 (55.6)	27 (45)
PS		
0	34 (94.4)	33 (55)
1–2	2 (5.6)	18 (30)
Not available	0 (0)	9 (15)
Histologic subtypes		
Leiomyosarcoma	8 (22.2)	13 (21.7)
Others	28 (77.8)	47 (78.3)
Tumor site		
Limbs/girdles	18 (50)	37 (61.7)
Others	18 (50)	23 (38.3)
Comorbidities (grade)		
G 1–2	25 (69.4)	41 (68.3)
G 3	2 (5.6)	5 (8.3)
G 4	0 (0)	0 (0)
Not available	9 (25)	14 (23.3)
Comorbidities (typology)		
Hypertension	13 (36.1)	29 (48.3)
Diabetes	3 (8.3)	6 (10)
Arrhythmias	1 (2.8)	6 (10)
Obesity	7 (19.4)	7 (11.7)
History of ischemic heart disease	3 (8.3)	4 (6.7)
Dyslipidemia	9 (25)	4 (6.7)
Smoke (with ex-smokers)	9 (25)	10 (16.7)
Center for surgery		
IOV/other referral centers	8 (22.2)	17 (28.3)
Peripheral centers	7 (19.4)	14 (23.3)
Not available	21 (58.4)	29 (48.4)
T		
1	4 (11.1)	0 (0)
2	31 (86.1)	60 (100)
Not available	1 (2.8)	0 (0)
Grade		
2	2 (5.6)	1 (1.7)
3	34 (94.4)	59 (98.3)
Stage		
II	2 (5.6)	0 (0)
III	34 (94.4)	60 (100)
Infiltrated margins		
Yes	9 (25)	23 (38.3)
No	20 (55.6)	26 (43.3)
Not available	7 (19.4)	11 (18.4)
Radiotherapy		
Yes	21 (58.3)	31 (51.7)
No	15 (41.7)	29 (48.3)
Not available	18 (50)	27 (45)
Metastasis		
Yes	14 (38.9)	38 (63.3)
No	22 (61.1)	22 (36.7)

In the ACT group median age was 49 years (range 19–75 years), baseline PS was 0 or 1, respectively, in 34 and two patients. Twenty-seven patients (75 %) had associated diseases, mostly low grade according to CIRS (Linn et al. 1968), three grade 3 and no grade 4 (Table 1). No patient had abnormal echocardiogram of the 27 examined. Out of three patients with a history of ischemic cardiomyopathy, two were treated with pegylated liposomal doxorubicin and one with conventional therapy (Table 2).

**Table 2:** Adjuvant chemotherapy regimens

Schedule (each 3 weeks)	No. of patients
Pegylated liposomal doxorubicin: 50 mg/mq day 1 q28	1
Pegylated liposomal doxorubicin: 30 mg/mq day 1 Ifosfamide: 3 g/mq days 1, 2, 3	1
Doxorubicin 37.5 mg/mq days 1, 2 Ifosfamide: 3 g/mq days 1, 2, 3	2
Epirubicin: 60 mg/mq days 1, 2 Ifosfamide: 3 g/mq days 1, 2, 3 or 1.8 g/mq days 1–5	32

In the NACT group, median age was 66 years (range 22–88 years), pts overall displayed a higher degree of frailty, with PS being 0 in 33, 1 in 16 and 2 in two patients. Forty-six patients (76.6 %) presented some comorbidity (Table 1). Thirty-nine patients (65 %) had cardiovascular risk factors, with some degree of impairment at the echocardiogram in three subjects (5 %).

### Efficacy

**Fig. 1:**
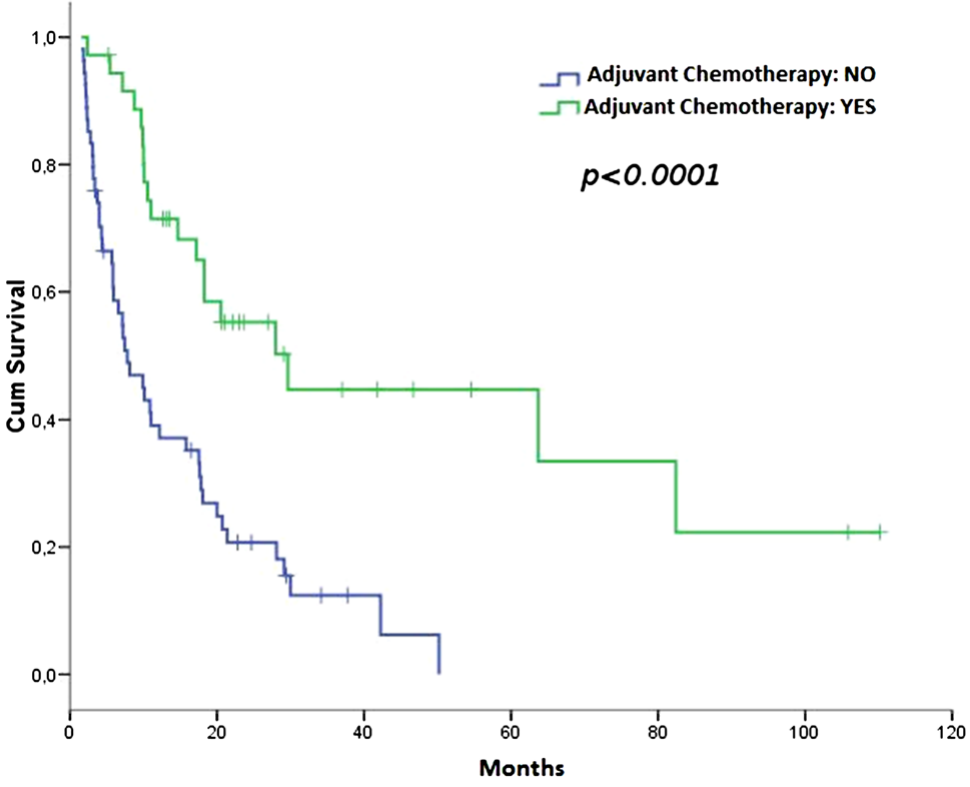
DFS according to adjuvant chemotherapy (ACT vs. NACT)

After a median follow-up of 28.2 months (range 3.4–114.3), disease recurrence was observed in 19 patients in the ACT group, and in 45 patients in the NACT group. The median DFS was 29.6 months in the ACT (95 % CI 13.2–46.0) and 7.8 months in the NACT group (95 % CI 3.9–11.7), HR 0.32 (95 % CI 0.183–0.565); (*p* < 0.0001; Fig. 1).

**Fig. 2:**
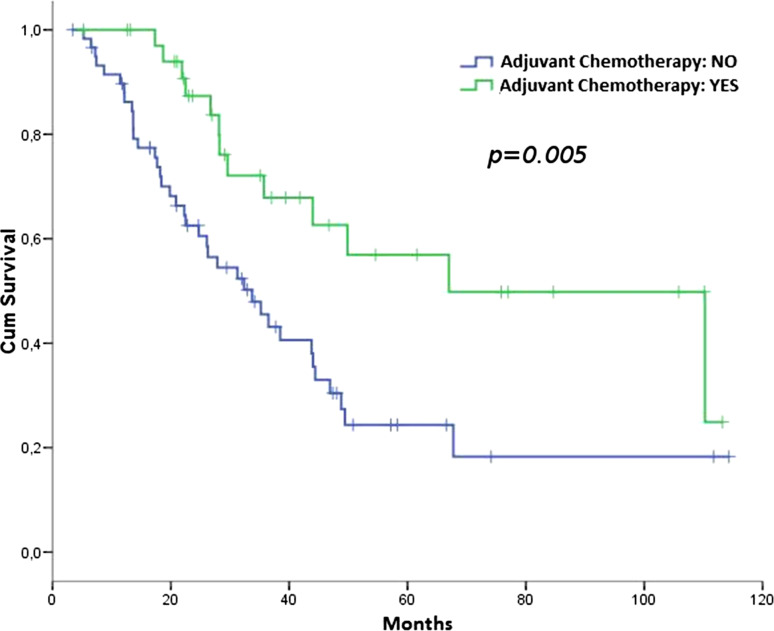
OS according to adjuvant chemotherapy (ACT vs. NACT)

Overall 51 patients died, 13 in the ACT group and 38 in the NACT group, of which 37 were attributable to sarcoma—11 in the ACT group and 26 in the NACT group. The median OS was 67.0 months in the ACT group (95 % CI 25.4–108.6) and 33.7 in the NACT group (95 % CI 23.3–44.2), HR 0.41 (95 % CI 0.219–0.779); (*p* = 0.005; Fig. 2).

When considering for the survival analysis of only patients who died of disease, a significant difference was observed between the two groups, with median OS of 29.6 months in the ACT group and 18.0 months in the NACT group; *p* = 0.008.

**Fig. 3:**
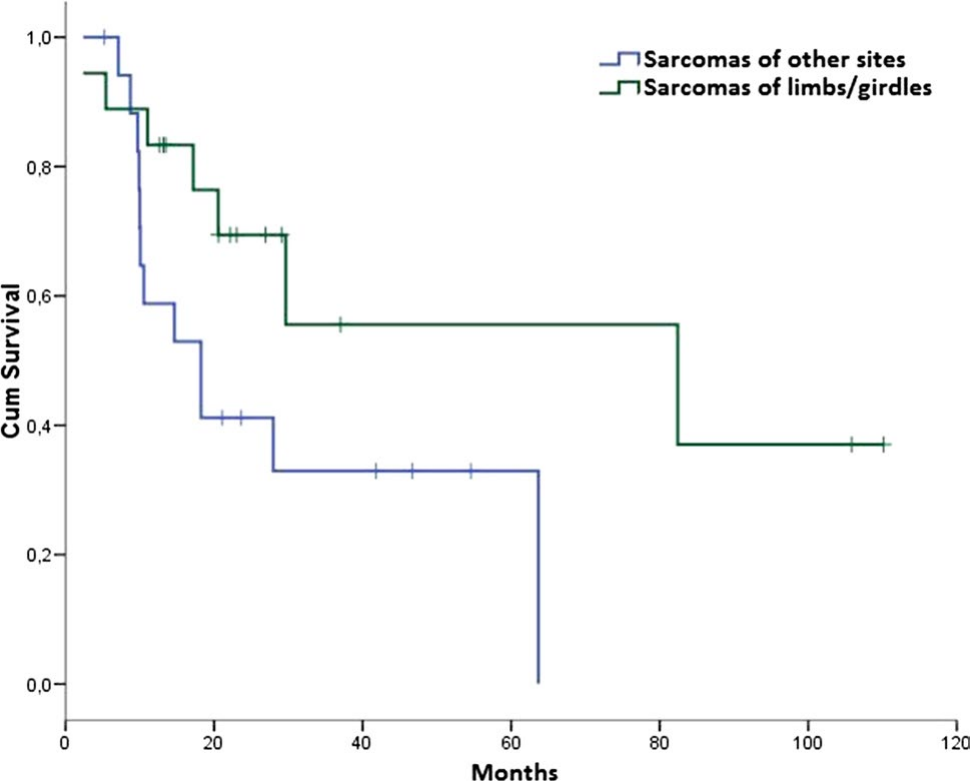
DFS in the ACT group according to the primary tumor site

Globally, median DFS for patients with limb/girdle STS was 82.4 months (95 % CI 0.0–184.7) versus 18.3 months (95 % CI 8.0–28.5) for patients with STS of other sites, *p* = 0.052 (Fig. 3).

A significant difference was observed for OS, but not for DFS, in patients with PS 0 compared to patients with PS 1–2, both in the ACT group (median OS 110.3 vs. 21.9 months; *p* = 0.012) and in the NACT group (median OS 44.0 vs. 17.7 months; *p* < 0.001).

No difference was found according to other factors (i.e., age, tumor size, deliver of radiotherapy, gender).

For patients in the ACT group, mean number of (neo)adjuvant chemotherapy courses delivered was five. Thirty patients (83.3 %) received more than three courses, and six patients (16.7 %) received three or less courses. Disease recurrence did occur in 17 patients receiving more than three courses, and in two patients receiving three or less courses. With regard to the number of chemotherapy cycles administered, no difference in survival was observed.

Dose intensity (DI), defined as the amount of drug delivered per time unit (mg/m^2^ for week), was calculated for patients in the ACT group. Nine patients (29 %) needed a dose reduction and five patients (16 %) a dose delay, with 26 patients (72.2 %) receiving a mean DI ≥ 85 %.

### Toxicity

Severe (grade 3–4 according to CTCAE v3.0) non-hematological and hematological toxicities occurred in 20 patients (55.6 %), leading to dose reductions, dose delays, and treatment discontinuation in five cases (13.9 %). There was no treatment-related death. Thirty-one patients (86.1 %) experienced hematological toxicity, with anemia being present in 80.5 %, neutropenia in 58.3 %, and thrombocytopenia in 36.1 % of patients (Table 3). Ten patients (27.7 %) experienced febrile neutropenia, successfully managed with no further complications. Non-hematological toxicity occurred in 30 patients (83.3 %), the most common being nausea/vomiting (58.3 %), fatigue (19.4 %), mucositis (11.1 %), other gastrointestinal toxicities (diarrhea, constipation etc.) (13.9 %), and other less frequent toxicities (52.8 %) (i.e., thromboembolic events, infections) (Table 4).

**Table 3:** Hematological toxicities (maximum grade)

Toxicities	Grade 1	Grade 2	Grade 3	Grade 4
Neutropenia	6	1	2	14
Febrile neutropenia	NA	NA	NA	10
Anemia	12	10	6	1
Thrombocytopenia	4	3	5	1

*NA* not applicable

**Table 4:** Non-hematological toxicities (maximum grade)

Toxicities	Grade 1	Grade 2	Grade 3	Grade 4
Nausea/vomiting	9	10	2	0
Mucositis	0	4	0	0
Fatigue	5	1	1	0
H&F syndrome	0	1	0	0
Other GI	4	0	0	1
Cardiotoxicity	1	0	0	0
Neuropathy c/p	1	2	1	0
Other toxicities	16	8	3	1

*H&F* hand and foot, *GI* gastrointestinal

## Discussion

Adjuvant treatment of localized STS is still a debated issue (ESMO 2012; Comandone 2014). With all the limits and cautions of a retrospective study, our study provides real-life data on the efficacy and tolerability of adjuvant chemotherapy.

Given the rarity of the disease, a cohort of 96 patients can be considered a reliable sample that is likely representative of the patient normally seen in routine clinical practice. In our study, in order to minimize possible disease-related biases for group comparison, we considered for inclusion only those patients with radically resected sarcoma at high risk of relapse. Indeed, eventual benefits of adjuvant chemotherapy seem likely to be limited to high-grade STS, as a recent analysis of more than 3200 patients included in the French Sarcoma Group database shows (Italiano et al. 2014). Such an advantage has been found in another recently published mono-institutional retrospective study (Schenone et al. 2014).

Nevertheless, as expected, the NACT group included on average older patients, with more than a half being 65 years or older, compared to less than a fifth in the ACT group, and less performing patients, with 30 % of patients in the NACT having ECOG PS 1–2 versus 5.6 % in the ACT group. Comorbidity did not differ in the two groups.

Our study demonstrated a benefit provided by adjuvant chemotherapy, with regard to both DFS and OS. One may argue that OS in patients not undergoing adjuvant chemotherapy may be shorter due to concomitant disease and older age, yet the benefit in OS for patients in the ACT group holds true even if we exclude patients who died because of unknown causes. Survival analysis comparing patients with an age cutoff of 65 years in both groups did not show any significant statistical difference neither for DFS nor for OS, whereas patients with PS 0 had a better survival both in the treated group and in not treated group.

Moreover, even if OS may indeed be influenced by a higher prevalence of frail patients in the NACT group, this condition does not factor in for DFS, which mainly depends on the characteristics of the disease that are homogeneous in both groups. Also, even if the majority of the patients have stage III disease both in the NACT and ACT group, patients having tumor stages II and III are not identical between the two groups, and this should at least in part be taken into account for the great variability in DFS (29.6 vs. 7.8 months).

Median survival, both recurrence-free survival and overall survival, in our study is shorter than observed in randomized trials, yet it must be acknowledged that by eligibility criteria we included only patients with high-grade STS, in order to keep the two cohorts of patients as homogenous as possible. Grade is indeed one of the strongest prognostic factors in STS. The EORTC 62931 trial (Woll et al. 2012), in which median DFS was 90.6 months and median OS was 148.8 months, included a not-negligible portion of patients with low or intermediate grade sarcomas (7 and 49 % respectively) that are characterized by better outcomes. Moreover, in our cohort, only roughly one-fourth of patients had surgery performed in a referral center, factor that is known to be crucial for survival (ESMO guidelines 2012; Rossi et al. 2013; Ray-Coquard et al. 2004; Clasby et al. 1997). Also, only a half of the patients in our study had STS located in the limbs or girdles, sites where adjuvant chemotherapy has shown a better activity (SMAC 1997). Indeed, when we consider only the patients with neoplasm of extremities, the median DFS is 82.4 months, thus comparable to literature data.

Among the factors that may influence the impact of chemotherapy on survival is dose intensity. In the update of the Italian Sarcoma Group trial (Frustaci et al. 2003), the benefit in OS was lost at a longer follow-up of 89.6 months (*p* = 0.07), but when considering only patients receiving DI ≥ 85 % the trend in favor of chemotherapy is maintained (*p* = 0.034). In our study 72 % of the patients in the ACT group received a DI ≥ 85 %, and this must be taken into account for the advantage we found from adjuvant chemotherapy.

Also, more than 80 % of our patients received more than three cycles of adjuvant chemotherapy, and we did not find any difference in outcomes compared to those patients receiving three cycles or less. This is likely due to the small number of subjects who received less than three cycles; nonetheless, such results are consistent with those reported by Gronchi and colleagues (Gronchi et al. 2012) showing non-inferiority of three cycles of chemotherapy compared to five cycles.

As for the tolerability and treatment-related toxicity, 55 % of the patients in the chemotherapy group experienced grade 3–4 toxicities. In five cases, toxicity led to early interruption of therapy. Anemia was the most frequent hematological toxicity, with neutropenia being the second most frequent hematological toxicity, present in about a half of the patients and with cases of febrile neutropenia successfully managed with no major complications. These results are consistent with the study of Frustaci and colleagues, where 58 % of patients developed severe neutropenia, whereas in the study by Gronchi et al. severe neutropenia was observed in 77 % of patients who received three cycles of chemotherapy and in 83 % of those who received five cycles.

As for non-hematological toxicities, the most frequently reported were nausea/vomiting, which were mainly low grade, present in two-thirds of patients. There was only a slight and asymptomatic reduction in left ventricular ejection fraction in one patient, without other cardiac toxicities. It must be observed that patients were selected for suitability to anthracyclines and pegylated liposomal doxorubicin was used instead of conventional anthracycline in two cases.

Globally, in our experience, treatment with anthracycline and ifosfamide despite its toxicity was feasible with no major side effects and no serious cardiac events.

## Conclusion

Even with the limits given by the retrospective nature of the study, our results confirm that adjuvant chemotherapy with anthracycline and ifosfamide after definitive surgery confers benefits in terms of DFS and OS in patients with high-risk sarcoma, namely deep-seated sarcomas which are larger than 5 cm and high grade. The greatest benefit is seen for patients with sarcomas of the extremities.

Given the high rate of hematological toxicity, and considering our results that are consistent with published data (Gronchi et al. 2012), three cycles of adjuvant chemotherapy with anthracycline and ifosfamide in full doses, with adequate support, may be recommended in selected patients with high risk radically resected STS.
